# Molecular Characterization of Spontaneous Mesenchymal Stem Cell Transformation

**DOI:** 10.1371/journal.pone.0001398

**Published:** 2008-01-02

**Authors:** Daniel Rubio, Silvia Garcia, Maria F. Paz, Teresa De la Cueva, Luis A. Lopez-Fernandez, Alison C. Lloyd, Javier Garcia-Castro, Antonio Bernad

**Affiliations:** 1 Department of Immunology and Oncology, Centro Nacional de Biotecnología, Consejo Superior de Investigaciones Científicas (CSIC), Universidad Autónoma de Madrid, Madrid, Spain; 2 Servicio de Farmacia, Hospital General Universitario Gregorio Marañón, Madrid, Spain; 3 Laboratory for Molecular Cell Biology, University College London, London, United Kingdom; 4 Andalusian Stem Cell Bank, Granada, Spain; City of Hope Medical Center, United States of America

## Abstract

**Background:**

We previously reported the *in vitro* spontaneous transformation of human mesenchymal stem cells (MSC) generating a population with tumorigenic potential, that we termed transformed mesenchymal cells (TMC).

**Methodology/Principal Findings:**

Here we have characterized the molecular changes associated with TMC generation. Using microarrays techniques we identified a set of altered pathways and a greater number of downregulated than upregulated genes during MSC transformation, in part due to the expression of many untranslated RNAs in MSC. Microarray results were validated by qRT-PCR and protein detection.

**Conclusions/Significance:**

In our model, the transformation process takes place through two sequential steps; first MSC bypass senescence by upregulating c-myc and repressing p16 levels. The cells then bypass cell crisis with acquisition of telomerase activity, Ink4a/Arf locus deletion and Rb hyperphosphorylation. Other transformation-associated changes include modulation of mitochondrial metabolism, DNA damage-repair proteins and cell cycle regulators. In this work we have characterized the molecular mechanisms implicated in TMC generation and we propose a two-stage model by which a human MSC becomes a tumor cell.

## Introduction

The development of a solid tumor is considered a multi-step process in which several molecular checkpoints must be altered to generate a tumor from a normal cell [1]. The acquired capabilities of tumor cells include their ability to proliferate continuously ignoring apoptosis or growth-inhibitory signals, generating their own mitogenic signals. In advanced phases of tumor development, a neoangiogenesis process takes place and finally tumor cells acquire the capacity of tissue invasion and metastasize to other organs. Generally, it is admitted that most tumors acquire these characteristics through genome instability, telomere stabilization and disruption of regulatory circuits [2].

A recent theory suggests the existence of cancer stem cells (CSC), a subpopulation of cells with tumorigenic potential that is lacked in the rest of the cells within this tumor. CSC were reported for some tumor types including breast and lung cancer, leukemia and glioblastoma [3], [4]. However, there is a great ignorance about how the “acquired capabilities” of tumor cells would take place; directly on adult stem cells, or on differentiated cells that suffer a dedifferentiation process. In this regard, CSC share several features with adult stem cells such as self-renewal ability, asymmetric division, and differentiation potential [5].

Adult human mesenchymal stem cell spontaneous immortalization and transformation were recently reported by our group [6], supporting the hypothesis of the stem cell origin of CSC. Independent laboratories have confirmed these data, reporting similar results using MSC derived from human or murine bone marrow [7]–[11]. In this regard, we have previously characterized the cellular sequence of steps necessary to transform a human MSC into a tumorigenic cell [6]. Following approximately 20 population doublings *in vitro*, mesenchymal stem cell cultures enter a senescence phase, but are able to bypass it at a high frequency. These cells then continue to divide until they reach a crisis phase. Only some samples are able to escape from this crisis phase spontaneously, but those that do have undergone tumorigenic transformation generating TMC.

However, until now genetic alterations implied in spontaneous MSC transformation are little known. Some groups have studied molecular pathways involved in the artificial transformation of MSC transduced with oncogenes [12], [13]. In this study, we have characterized the molecular mechanisms implicated in TMC generation and we propose a two-stage model by which a human MSC becomes *in vitro* a tumor cell.

## Results

### Comparative gene expression analysis of MSC transformation by microarray analysis

To analyze molecular differences associated with TMC generation, we performed microarray studies using mRNA from pre- and post-senescence MSC, and from TMC. From a general perspective, data analysis showed that the greatest changes were associated with TMC generation, as TMC functions were more different than post-senescence MSC, compared to pre-senescence MSC (Table 1). Although in a minor intensity, post-senescence MSC have the same altered functions that TMC. In both cases the principal category affected is “cancer” (Table 1). However, the main pathways deregulated in both, post-senescence MSC and TMC, are related to stress, toxic events and mitochondrial metabolism (Table 2). On the other hand, there was more down- than upregulated RNA transcripts associated with TMC generation (Figure S1). The main differences in mRNA expression profiles between pre- and post-senescence MSC are shown in Table 3. Table 4 shows differences between pre-senescence MSC and TMC.

**Table 1:** Comparative table of functions with a higher significance in selected genes for post-senescence MSC and TMC, obtained by Ingenuity Pathways Analysis software.

Category	Process Annotation	pre-sen/post-sen MSC		pre-sen MSC/TMC	
		Significance	Molecules	Significance	Molecules
Cancer	Tumorigenesis	2.73E-18	22	4.71E-27	41
Cellular Movement	Migration of eukaryotic cells	4.38E-07	9	1.74E-18	25
Tissue Development	Developmental process of tissue	1.35E-07	9	1.35E-17	23
Gastrointestinal Disease	Colorectal cancer	9.32E-12	9	1.75E-17	16
Genetic Disorder	Genetic disorder	2.40E-05	8	2.74E-17	26
Cell Death	Cell death of eukaryotic cells	3.06E-11	16	6.73E-20	34
Organismal Survival	Survival of mice	3.14E-08	7	N/A	N/A
Organismal Survival	Death of mammalia	1.84E-04	6	3.62E-11	17
Neurological Disease	Cell death of neurons	4.71E-08	7	2.30E-05	7

In each category only the highest “Process Annotation” is represented.

**Table 2:** Top tox list obtained by Ingenuity Pathways Analysis for post-senescence MSC and TMC.

MSC presen/MSCpost-sen	p-value	Ratio
Aryl Hydrocarbon Receptor Signaling	1.97E_05	4/154 (0.026)
Hepatic fibrosis	3.48E_03	2/85 (0.024)
Xenobiotic Metabolism	7.83E_03	2/129 (0.016)
Cytochrome P450 Panel	1.43E_02	1/14 (0.071)

MSC presen/TMC	p-value	Ratio
Hepatic fibrosis	2.11E_14	10/85 (0.118)
FXF/RXR activation	1.18E_03	2/20 (0.100)
Mitochondrial dysfunction	4.70E_03	3/125 (0.024)
Hepatic cholestasis	5.04E_03	3/125 (0.022)
LPS & IL-1 mediated inhibition of RXR function	1.19E_02	3/185 (0.016)

**Table 3:** Main mRNA differences between pre- and post-senescence MSC.

Genbank Acc No	Gene name	x-fold change	p-value	z-score
NM_004932	Cadherin 6, type 2, K-cadherin (fetal kidney)	−3.675	<0.001	−8.612
AL049227	Homo sapiens mRNA; cDNA DKFZp564N1116 (from clone DKFZp564N1116)	−3.173	<0.001	−8.827
NM_021197	WAP four-disulfide core domain 1	−3.151	0.005	−8.772
NM_003012	Secreted frizzled-related protein 1	−3.048	0.001	−6.866
AF070524	Homo sapiens clone 24453 mRNA sequence	−2.664	0.006	−6.775
AB046796	KIAA1576 protein	−2.346	0.004	−4.803
NM_013381	Thyrotropin-releasing hormone degrading ectoenzyme	−2.272	0.004	−5.253
AK022997	Homo sapiens cDNA FLJ12935 fis. clone NT2RP2004982	−2.265	0.003	−5.048
NM_006208	Ectonucleotide pyrophosphatase/phosphodiesterase 1	−2.259	0.003	−4.791
NM_033211	Hypothetical gene supported by AF038182; BC009203	−2.220	0.001	−4.689
AJ279081	Chromosome 21 open reading frame 66	−2.188	<0.001	−4.726
NM_006389	Oxygen regulated protein (150kD)	−2.173	0.009	−4.402
NM_000627	Latent transforming growth factor beta binding protein 1	−2.094	<0.001	−4.554
NM_001271	Chromodomain helicase DNA binding protein 2	−2.076	0.006	−6.785
NM_002937	Ribonuclease. RNase A family. 4	−2.016	<0.001	−3.940
NM_000325	Paired-like homeodomain transcription factor 2	−1.959	<0.001	−3.954
NM_000930	Plasminogen activator. tissue	−1.930	0.001	−4.655
AK057782	Homo sapiens cDNA FLJ25053 fis. clone CBL04266	−1.908	0.002	−5.726
NM_005382	Neurofilament 3 (150kD medium)	−1.884	0.001	−3.872
U67784	G protein-coupled receptor	−1.855	<0.001	−3.732
NM_021730	Hypothetical protein PP1044	−1.833	0.002	−3.514
NM_002977	Sodium channel. voltage-gated. type IX. alpha polypeptide	−1.823	0.000	−3.512
NM_032883	Chromosome 20 open reading frame 100	−1.804	0.005	−3.561
NM_004405	Distal-less homeo box 2	−1.788	0.009	−4.443
AF263545	Homo sapiens HUT11 protein mRNA. partial 3′ UTR	−1.776	0.007	−5.334
D13628	Angiopoietin 1	−1.748	<0.001	−4.393
NM_031957	Keratin associated protein 1.5	−1.738	0.001	−4.048
BC012486	Keratin associated protein KRTAP2.1A	−1.732	<0.001	−3.517
NM_021226	Hypothetical protein from clones 23549 and 23762	−1.694	0.007	−3.864
NM_000077	Cyclin-dependent kinase inhibitor 2A (melanoma. p16. inhibits CDK4)	−1.686	0.003	−4.109
AF070641	Homo sapiens clone 24421 mRNA sequence	−1.660	0.007	−4.773
NM_004288	Pleckstrin homology. Sec7 and coiled/coil domains. binding protein	−1.636	0.009	−4.299
NM_018476	Brain expressed. X-linked 1	−1.524	<0.001	−3.971
AF161403	Homo sapiens HSPC285 mRNA. partial cds	1.507	0.001	3.846
NM_019111	Major histocompatibility complex. class II. DR alpha	1.598	<0.001	3.582
AP001660	Homo sapiens genomic DNA. chromosome 21q. section 4/105	1.654	0.003	4.720
AK022034	Homo sapiens cDNA FLJ11972 fis. clone HEMBB1001209	1.667	0.008	3.744
NM_005435	Rho guanine nucleotide exchange factor (GEF) 5	1.687	<0.001	4.854
NM_024081	Transmembrane gamma-carboxyglutamic acid protein 4	1.733	<0.001	4.446
NM_004938	Death-associated protein kinase 1	1.776	0.008	5.389
NM_004962	Growth differentiation factor 10	1.817	0.004	5.410
NM_000900	Matrix Gla protein	1.828	0.003	5.265
NM_012068	Activating transcription factor 5	1.832	0.002	3.701
NM_014424	Heat shock 27kD protein family. member 7 (cardiovascular)	1.855	0.004	3.603
NM_022719	DiGeorge syndrome critical region gene DGSI; likely ortholog of mouse expressed sequence 2 embryonic	1.879	0.003	3.887
NM_004669	Chloride intracellular channel 3	1.889	0.006	4.662
NM_001673	Asparagine synthetase	1.896	0.006	3.594
NM_000095	Cartilage oligomeric matrix protein (pseudoachondroplasia. epiphyseal dysplasia 1. multiple)	1.921	<0.001	3.665
NM_001353	Aldo-keto reductase family 1. member C1 (dihydrodiol dehydrogenase 1; 20-alpha (3-alpha)-hydroxyster	1.947	<0.001	3.742
NM_019105	Tenascin XB	2.036	0.001	4.471
AB046843	KIAA1623 protein	2.040	0.001	5.225
AF111170	Homo sapiens 14q32 Jagged2 gene. complete cds; and unknown gene	2.051	<0.001	4.030
NM_007223	Putative G protein coupled receptor	2.126	0.006	6.832
AK057721	Homo sapiens cDNA FLJ33159 fis. clone UTERU2000465	2.134	0.002	5.097
NM_003480	Microfibril-associated glycoprotein-2	2.307	0.002	4.891
AK024396	Acetyl-Coenzyme A synthetase 2 (AMP forming)-like	2.330	0.002	6.343
BC015794	Hypothetical protein FLJ10097	2.367	<0.001	5.142
AK024428	Pleckstrin homology. Sec7 and coiled/coil domains 4	2.415	0.007	6.613
AK024240	Homo sapiens cDNA FLJ14178 fis. clone NT2RP2003339	2.451	0.001	6.199
NM_005264	GDNF family receptor alpha 1	2.465	<0.001	6.065
L48728	Homo sapiens T cell receptor beta (TCRBV10S1) gene. complete cds	2.626	0.004	7.592
NM_005556	Keratin 7	2.795	0.007	5.917
NM_007281	Scrapie responsive protein 1	2.828	<0.001	6.276
NM_005596	Nuclear factor I/B	2.844	<0.001	6.389
NM_004750	Cytokine receptor-like factor 1	3.027	<0.001	6.610
AK055188	Homo sapiens cDNA FLJ30626 fis. clone CTONG2001911. weakly similar to UBIQUITIN CARBOXYL-TERMINAL	3.340	0.002	7.033
AF311912	Secreted frizzled-related protein 2	3.365	0.001	7.630
AL080218	Homo sapiens mRNA; cDNA DKFZp586N1323 (from clone DKFZp586N1323)	3.422	0.002	7.428
U14383	Mucin 8. tracheobronchial	3.791	0.004	7.836
NM_000095	Cartilage oligomeric matrix protein (pseudoachondroplasia. epiphyseal dysplasia 1. multiple)	3.796	0.003	7.483
AK000819	Homo sapiens cDNA FLJ20812 fis. clone ADSE01316	3.953	<0.001	8.800
NM_015863	Surfactant protein B	4.343	<0.001	13.305
NM_005532	Interferon. alpha-inducible protein 27	5.236	<0.001	12.417

Array data were filtered according to Z-score (>3.5 and <−3.5) and p-value (<0.01).

**Table 4:** Main mRNA differences between pre-senescence MSC and TMC.

Genbank Acc No.	Gene name	x-fold change	p-value	z-score
NM_000165	Gap junction protein, alpha 1, 43kD (connexin 43)	−52.904	<0.001	−6.640
BC014245	Homo sapiens, Similar to RIKEN cDNA 1110014B07 gene, clone MGC:20766 IMAGE:4586039, mRNA, complete c	−45.496	<0.001	−6.311
NM_000089	Collagen, type I, alpha 2	−44.113	<0.001	−6.041
NM_002421	Matrix metalloproteinase 1 (interstitial collagenase)	−31.671	<0.001	−5.512
NM_006475	Osteoblast specific factor 2 (fasciclin I-like)	−31.206	<0.001	−5.513
NM_052947	Heart alpha-kinase	−30.941	<0.001	−5.499
NM_000089	Collagen, type I, alpha 2	−30.183	<0.001	−5.443
NM_002937	Ribonuclease, RNase A family, 4	−30.113	<0.001	−5.911
NM_013372	Cysteine knot superfamily 1, BMP antagonist 1	−27.678	<0.001	−5.301
NM_006475	Osteoblast specific factor 2 (fasciclin I-like)	−25.377	<0.001	−5.225
NM_006063	Sarcomeric muscle protein	−25.291	<0.001	−5.502
M96843	Striated muscle contraction regulatory protein	−24.615	<0.001	−5.414
NM_002526	5′ nucleotidase (CD73)	−24.069	<0.001	−5.258
NM_021242	Hypothetical protein STRAIT11499	−22.708	<0.001	−6.307
AK027274	Homo sapiens cDNA FLJ14368 fis, clone HEMBA1001122	−21.750	<0.001	−7.398
AB033025	KIAA1199 protein	−21.257	<0.001	−4.880
NM_032348	Hypothetical protein MGC3047	−20.763	<0.001	−4.840
NM_000963	Prostaglandin-endoperoxide synthase 2 (prostaglandin G/H synthase and cyclooxygenase)	−20.328	<0.001	−5.091
AB058761	KIAA1858 protein	−20.016	<0.001	−5.644
NM_006211	Proenkephalin	−19.361	<0.001	−4.731
AK055725	Maternally expressed 3	−19.350	<0.001	−4.747
NM_000138	Fibrillin 1 (Marfan syndrome)	−18.750	<0.001	−4.679
AB011145	KIAA0573 protein	−18.677	<0.001	−5.912
NM_006988	A disintegrin-like and metalloprotease (reprolysin type) with thrombospondin type 1 motif, 1	−18.388	<0.001	−4.649
AB014511	ATPase, Class II, type 9A	−17.675	<0.001	−4.701
NM_000393	Collagen, type V, alpha 2	−17.533	<0.001	−4.628
NM_000419	Integrin, alpha 2b (platelet glycoprotein IIb of IIb/IIIa complex, antigen CD41B)	−17.439	<0.001	−5.061
NM_007268	Ig superfamily protein	−17.320	<0.001	−4.951
NM_015696	Weakly similar to glutathione peroxidase 2	−16.943	<0.001	−4.525
NM_016651	Heptacellular carcinoma novel gene-3 protein	−16.524	<0.001	−4.966
NM_002593	Procollagen C-endopeptidase enhancer	−16.332	<0.001	−4.476
NM_000358	Transforming growth factor, beta-induced, 68kD	−16.120	<0.001	−4.438
NM_031442	Brain cell membrane protein 1	−15.709	<0.001	−4.508
NM_015364	MD-2 protein	−15.455	<0.001	−4.753
NM_002487	Necdin homolog (mouse)	−15.122	<0.001	−5.116
AF109681	Integrin, alpha 11	−15.057	<0.001	−4.708
NM_000916	Oxytocin receptor	−14.278	<0.001	−4.260
AL136693	Duodenal cytochrome b	−14.265	<0.001	−4.350
AK055976	Thymosin, beta 4, X chromosome	−14.159	<0.001	−4.233
AK025931	Homo sapiens cDNA: FLJ22278 fis, clone HRC03745	−13.834	<0.001	−4.949
BC000257	Homo sapiens, clone IMAGE:3357862, mRNA, partial cds	−13.804	<0.001	−4.193
NM_007085	Follistatin-like 1	−13.508	<0.001	−4.164
NM_022726	Elongation of very long chain fatty acids (FEN1/Elo2, SUR4/Elo3, yeast)-like 4	−12.892	<0.001	−4.526
NM_005847	Solute carrier family 23 (nucleobase transporters), member 2	−12.779	<0.001	−4.511
NM_030781	Collectin sub-family member 12	−12.550	<0.001	−5.284
NM_000093	Collagen, type V, alpha 1	−12.506	<0.001	−4.033
NM_001353	Aldo-keto reductase family 1, member C1 (dihydrodiol dehydrogenase 1; 20-alpha (3-alpha)-hydroxyster	−12.332	<0.001	−4.011
NM_002064	Glutaredoxin (thioltransferase)	−12.314	<0.001	−4.005
NM_020404	Tumor endothelial marker 1 precursor	−12.138	<0.001	−4.086
NM_000962	Prostaglandin-endoperoxide synthase 1 (prostaglandin G/H synthase and cyclooxygenase)	−11.886	<0.001	−4.382
NM_022360	Human epididymis-specific 3 beta	−11.816	0.001	−4.766
BC009078	Homo sapiens, clone MGC:17624 IMAGE:3855543, mRNA, complete cds	−11.792	<0.001	−4.284
AB051443	KIAA1656 protein	−11.742	<0.001	−7.230
NM_031440	Transmembrane protein 7	−11.686	<0.001	−5.635
NM_002421	Matrix metalloproteinase 1 (interstitial collagenase)	−11.641	<0.001	−3.918
NM_024031	Hypothetical protein MGC3121	−11.606	<0.001	−3.916
AF200348	Melanoma associated gene	−11.516	<0.001	−3.901
NM_031426	Hypothetical protein FLJ12783	−11.244	<0.001	−3.923
AK055903	Homo sapiens cDNA: FLJ21592 fis, clone COL07036	−11.134	<0.001	−3.847
NM_012104	Beta-site APP-cleaving enzyme	−10.628	<0.001	−3.819
AL133640	Homo sapiens mRNA; cDNA DKFZp586C1021 (from clone DKFZp586C1021); partial cds	−10.618	<0.001	−4.345
U17077	BENE protein	−10.516	<0.001	−4.085
NM_024563	Hypothetical protein FLJ14054	−10.297	<0.001	−4.710
NM_004098	Empty spiracles homolog 2 (Drosophila)	−10.242	<0.001	−3.715
AK023413	Homo sapiens cDNA FLJ13351 fis, clone OVARC1002156, weakly similar to Danio rerio uridine kinase mRN	−10.209	<0.001	−4.692
NM_000090	Collagen, type III, alpha 1 (Ehlers-Danlos syndrome type IV, autosomal dominant)	−10.049	<0.001	−3.684
NM_003247	Thrombospondin 2	−9.958	<0.001	−3.667
NM_005086	Sarcospan (Kras oncogene-associated gene)	−9.955	<0.001	−4.800
NM_004265	Fatty acid desaturase 2	−9.904	<0.001	−3.660
NM_014244	A disintegrin-like and metalloprotease (reprolysin type) with thrombospondin type 1 motif, 2	−9.728	<0.001	−4.028
AF131817	Homo sapiens clone 25023 mRNA sequence	−9.691	<0.001	−4.916
AK054816	Ferritin, heavy polypeptide 1	−9.653	<0.001	−3.629
AL050370	Homo sapiens mRNA; cDNA DKFZp566C0546 (from clone DKFZp566C0546)	−9.494	0.001	−4.871
NM_003246	Thrombospondin 1	−9.217	<0.001	−3.635
NM_004660	DEAD/H (Asp-Glu-Ala-Asp/His) box polypeptide, Y chromosome	−9.185	<0.001	−3.666
NM_014678	KIAA0685 gene product	−9.118	<0.001	−3.584
NM_016588	Neuritin	−8.963	<0.001	−4.233
NM_001235	Serine (or cysteine) proteinase inhibitor, clade H (heat shock protein 47), member 2	−8.923	<0.001	−3.503
AK057865	Thy-1 cell surface antigen	−8.798	<0.001	−4.707
NM_023927	Hypothetical protein FLJ21313	−8.689	<0.001	−3.574
NM_013253	Dickkopf homolog 3 (Xenopus laevis)	−8.612	<0.001	−4.497
AF111170	Homo sapiens 14q32 Jagged2 gene, complete cds; and unknown gene	−8.553	<0.001	−3.800
AF334710	Homo sapiens pyruvate dehydrogenase kinase 4 mRNA, 3′ untranslated region, partial sequence	−8.534	0.001	−4.138
NM_022143	NAG14 protein	−8.469	<0.001	−3.575
NM_002422	Matrix metalloproteinase 3 (stromelysin 1, progelatinase)	−8.442	0.001	−3.777
AK055249	Homo sapiens cDNA FLJ30687 fis, clone FCBBF2000379	−8.415	<0.001	−3.917
AJ279081	Chromosome 21 open reading frame 66	−8.329	<0.001	−4.091
NM_001541	Heat shock 27kD protein 2	−8.279	<0.001	−3.537
NM_017680	Asporin (LRR class 1)	−8.279	<0.001	−5.816
NM_004418	Dual specificity phosphatase 2	−8.249	<0.001	−4.407
AL049227	Homo sapiens mRNA; cDNA DKFZp564N1116 (from clone DKFZp564N1116)	−8.230	<0.001	−4.831
NM_031897	Calcium channel, voltage-dependent, gamma subunit 6	−8.127	<0.001	−5.033
AY040094	Serine protease HTRA3	−7.690	<0.001	−4.676
NM_002761	Protamine 1	−7.636	<0.001	−3.924
NM_001850	Collagen, type VIII, alpha 1	−7.617	<0.001	−3.919
AB011538	Slit homolog 3 (Drosophila)	−7.569	<0.001	−3.813
AB033073	Similar to glucosamine-6-sulfatases	−7.473	0.001	−3.627
NM_003885	Cyclin-dependent kinase 5, regulatory subunit 1 (p35)	−7.415	<0.001	−4.046
NM_030786	Intermediate filament protein syncoilin	−7.325	<0.001	−3.751
NM_001864	Cytochrome c oxidase subunit VIIa polypeptide 1 (muscle)	−7.295	<0.001	−3.654
AK001058	Homo sapiens cDNA FLJ10196 fis, clone HEMBA1004776	−7.238	<0.001	−3.569
NM_001375	Deoxyribonuclease II, lysosomal	−7.202	<0.001	−3.719
NM_002414	Antigen identified by monoclonal antibodies 12E7, F21 and O13	−7.199	<0.001	−3.630
AL080135	Hypothetical protein DKFZp434I143	−7.174	<0.001	−4.265
BF680501	Putative membrane protein	−7.108	<0.001	−6.487
NM_017980	Hypothetical protein FLJ10044	−6.999	<0.001	−3.665
M68874	Phospholipase A2, group IVA (cytosolic, calcium-dependent)	−6.795	<0.001	−5.273
NM_006552	Lipophilin A (uteroglobin family member)	−6.550	<0.001	−3.540
AK054724	Homo sapiens cDNA FLJ30162 fis, clone BRACE2000565	−6.401	<0.001	−3.877
AK025015	Homo sapiens cDNA: FLJ21362 fis, clone COL02886	−6.389	<0.001	−3.580
NM_002776	Kallikrein 10	−6.375	<0.001	−3.741
AF380356	Homo sapiens PBDX mRNA, complete cds	−6.263	<0.001	−4.673
NM_004529	Myeloid/lymphoid or mixed-lineage leukemia (trithorax homolog, Drosophila); translocated to, 3	−6.221	0.003	−4.190
NM_025258	NG37 protein	−6.045	0.002	−4.951
BC016964	Homo sapiens, clone MGC:21621 IMAGE:4181577, mRNA, complete cds	−5.945	<0.001	−4.086
AK022198	Homo sapiens cDNA FLJ12136 fis, clone MAMMA1000312	−5.889	0.008	−4.879
AK056857	Homo sapiens cDNA FLJ32295 fis, clone PROST2001823, weakly similar to TRANSCRIPTION FACTOR SP1	−5.879	0.001	−4.255
NM_032514	Microtubule-associated protein 1 light chain 3 alpha	−5.830	<0.001	−4.491
AK024734	Homo sapiens cDNA: FLJ21081 fis, clone CAS02959	−5.816	<0.001	−4.229
BC017981	Homo sapiens, Similar to RIKEN cDNA 2700038C09 gene, clone MGC:24600 IMAGE:4245342, mRNA, complete c	−5.685	0.001	−3.761
AF220030	Tripartite motif-containing 6	−5.666	<0.001	−4.166
NM_001458	Filamin C, gamma (actin binding protein 280)	−5.649	<0.001	−3.748
AK025786	Homo sapiens cDNA: FLJ22133 fis, clone HEP20529	−5.544	<0.001	−3.707
NM_001935	Dipeptidylpeptidase IV (CD26, adenosine deaminase complexing protein 2)	−5.426	<0.001	−4.964
AF131851	Hypothetical protein	−5.282	<0.001	−5.808
NM_031957	Keratin associated protein 1.5	−5.278	<0.001	−3.813
AK057853	Homo sapiens cDNA FLJ25124 fis, clone CBR06414	−5.132	<0.001	−5.054
BC004224	Homo sapiens, clone MGC:4762 IMAGE:3537945, mRNA, complete cds	−5.044	<0.001	−4.122
NM_000451	Short stature homeobox	−4.956	<0.001	−3.845
U12767	Nuclear receptor subfamily 4, group A, member 3	−4.840	<0.001	−4.340
NM_006517	Solute carrier family 16 (monocarboxylic acid transporters), member 2 (putative transporter)	−4.815	<0.001	−4.004
NM_018692	Chromosome 20 open reading frame 17	−4.722	<0.001	−5.134
NM_005130	Heparin-binding growth factor binding protein	−4.683	0.004	−6.093
AK026141	Homo sapiens cDNA: FLJ22488 fis, clone HRC10948, highly similar to HSU79298 Human clone 23803 mRNA	−4.639	<0.001	−3.517
AK055391	Homo sapiens cDNA FLJ30829 fis, clone FEBRA2001790, highly similar to Xenopus laevis RRM-containing	−4.535	<0.001	−5.706
AL359052	Homo sapiens mRNA full length insert cDNA clone EUROIMAGE 1968422	−4.486	<0.001	−3.606
NM_003012	Secreted frizzled-related protein 1	−4.471	0.002	−3.815
BC015134	Homo sapiens, clone IMAGE:3934391, mRNA	−4.455	0.005	−4.111
AK055501	Homo sapiens cDNA FLJ30939 fis, clone FEBRA2007414	−4.436	<0.001	−3.795
AF000994	Ubiquitously transcribed tetratricopeptide repeat gene, Y chromosome	−4.392	<0.001	−4.574
NM_002089	GRO2 oncogene	−4.319	<0.001	−4.839
NM_018271	Hypothetical protein FLJ10916	−4.274	<0.001	−5.289
NM_006030	Calcium channel, voltage-dependent, alpha 2/delta subunit 2	−4.269	0.005	−5.657
NM_052969	Ribosomal protein L39-like	−4.156	<0.001	−3.920
NM_022773	Hypothetical protein FLJ12681	−4.146	<0.001	−4.175
NM_004753	Short-chain dehydrogenase/reductase 1	−4.134	<0.001	−4.386
D13628	Angiopoietin 1	−4.075	0.003	−5.116
NM_004675	Ras homolog gene family, member I	−4.027	0.001	−3.549
NM_001532	Solute carrier family 29 (nucleoside transporters), member 2	−4.009	<0.001	−3.537
U16306	Chondroitin sulfate proteoglycan 2 (versican)	−3.959	<0.001	−3.505
NM_024806	Hypothetical protein FLJ23554	−3.806	<0.001	−3.678
AF152529	Protocadherin gamma subfamily B, 8 pseudogene	−3.770	0.003	−4.390
NM_006383	DNA-dependent protein kinase catalytic subunit-interacting protein 2	−3.676	0.001	−4.543
NM_020169	Latexin protein	−3.670	<0.001	−3.817
AK055969	Homo sapiens cDNA FLJ31407 fis, clone NT2NE2000137	−3.494	0.006	−4.646
BC011406	Homo sapiens, clone MGC:9758 IMAGE:3855620, mRNA, complete cds	−3.281	<0.001	−3.672
NM_014553	LBP protein; likely ortholog of mouse CRTR-1	−3.266	0.001	−4.130
NM_006821	Peroxisomal long-chain acyl-coA thioesterase	−3.221	<0.001	−3.869
NM_005098	Musculin (activated B-cell factor-1)	−3.198	0.002	−4.057
AK022355	Homo sapiens cDNA FLJ12293 fis, clone MAMMA1001815	−3.178	0.004	−4.693
NM_003178	Synapsin II	−3.130	0.001	−3.982
U14383	Mucin 8, tracheobronchial	−3.043	0.009	−4.336
NM_004257	TGF beta receptor associated protein -1	−3.028	0.006	−4.330
AK021632	Homo sapiens cDNA FLJ11570 fis, clone HEMBA1003309	−2.987	0.009	−3.984
NM_002108	Histidine ammonia-lyase	−2.982	<0.001	−3.812
NM_032880	Hypothetical protein MGC15730	−2.956	0.001	−3.585
BC015160	Homo sapiens, clone IMAGE:3885940, mRNA, partial cds	−2.873	0.004	−3.919
AK055509	Homo sapiens cDNA FLJ30947 fis, clone FEBRA2007714	−2.829	<0.001	−3.925
D86964	Dedicator of cyto-kinesis 2	−2.801	0.002	−3.595
NM_000639	Tumor necrosis factor (ligand) superfamily, member 6	−2.731	0.005	−3.872
NM_004694	Solute carrier family 16 (monocarboxylic acid transporters), member 6	−2.681	0.005	−3.662
AB007964	KIAA0495	−2.677	0.001	−3.586
X68994	H.sapiens CREB gene, exon Y	−2.654	0.004	−3.554
NM_013227	Aggrecan 1 (chondroitin sulfate proteoglycan 1, large aggregating proteoglycan, antigen identified b	−2.628	0.007	−3.646
AF019226	Glioblastoma overexpressed	−2.411	0.006	−3.507
AK054905	Homo sapiens cDNA FLJ30343 fis, clone BRACE2007502	1.931	0.006	4.281
AF339768	Homo sapiens clone IMAGE:119716, mRNA sequence	2.544	0.004	3.639
NM_000361	Thrombomodulin	2.620	0.001	3.636
NM_006187	2′-5′-oligoadenylate synthetase 3 (100 kD)	2.628	0.007	3.724
AK025390	Homo sapiens cDNA: FLJ21737 fis, clone COLF3396	2.772	0.001	3.713
AF130074	Homo sapiens clone FLB9348 PRO2523 mRNA, complete cds	2.805	0.001	4.057
AB032962	KIAA1136 protein	2.838	0.001	4.111
AJ420570	Homo sapiens cDNA FLJ14752 fis, clone NT2RP3003071	2.870	<0.001	3.839
NM_014811	KIAA0649 gene product	2.873	0.007	4.113
NM_004171	Solute carrier family 1 (glial high affinity glutamate transporter), member 2	2.950	0.008	4.227
NM_013982	Neuregulin 2	2.985	0.001	3.982
NM_032808	Hypothetical protein FLJ14594	3.020	<0.001	4.172
AB002366	KIAA0368 protein	3.085	0.009	4.403
NM_018700	Tripartite motif-containing 36	3.129	0.001	4.306
BC003376	ELAV (embryonic lethal, abnormal vision, Drosophila)-like 1 (Hu antigen R)	3.172	<0.001	3.568
AK023283	Homo sapiens cDNA FLJ13221 fis, clone NT2RP4002075	3.221	<0.001	3.869
AK054766	Homo sapiens cDNA FLJ30204 fis, clone BRACE2001496	3.329	0.001	3.717
NM_002829	Protein tyrosine phosphatase, non-receptor type 3	3.429	<0.001	3.617
NM_001618	ADP-ribosyltransferase (NAD+; poly (ADP-ribose) polymerase)	3.444	0.001	4.504
NM_001445	Fatty acid binding protein 6, ileal (gastrotropin)	3.450	0.001	4.096
NM_024565	Hypothetical protein FLJ14166	3.528	0.002	3.896
L05148	Zeta-chain (TCR) associated protein kinase (70 kD)	3.599	<0.001	3.759
NM_005525	Hydroxysteroid (11-beta) dehydrogenase 1	3.601	<0.001	3.525
NM_016931	NADPH oxidase 4	3.657	0.004	4.289
AK057339	Actin like protein	3.693	<0.001	4.038
NM_024771	Hypothetical protein FLJ13848	3.881	0.001	5.345
NM_032047	UDP-GlcNAc:betaGal beta-1,3-N-acetylglucosaminyltransferase 5	4.172	0.002	4.415
NM_014962	BTB (POZ) domain containing 3	4.237	0.002	5.362
NM_017780	KIAA1416 protein	4.243	<0.001	3.977
AB018295	KIAA0752 protein	4.556	<0.001	5.016
NM_006622	Serum-inducible kinase	4.639	0.001	4.742
NM_003651	Cold shock domain protein A	4.961	<0.001	4.080
AF268419	Homo sapiens chondrosarcoma CSAG1c mRNA sequence	5.423	<0.001	3.659
AK055111	Homo sapiens cDNA FLJ30549 fis, clone BRAWH2001484, weakly similar to POLYPEPTIDE N-ACETYLGALACTOSAM	5.503	<0.001	3.909
NM_006597	Heat shock 70kD protein 8	5.664	0.001	3.622
NM_019844	Solute carrier family 21 (organic anion transporter), member 8	5.919	0.000	4.271
AB037727	Cask-interacting protein 1	7.351	<0.001	3.597
AL137311	Homo sapiens mRNA; cDNA DKFZp761G02121 (from clone DKFZp761G02121); partial cds	8.238	<0.001	3.661
BC014584	Homo sapiens, clone IMAGE:4047062, mRNA	8.280	0.001	3.982
NM_003785	G antigen, family B, 1 (prostate associated)	8.326	<0.001	3.582
NM_005010	Neuronal cell adhesion molecule	8.533	<0.001	4.478
NM_033642	Fibroblast growth factor 13	9.666	<0.001	4.016
NM_018476	Brain expressed, X-linked 1	11.140	<0.001	3.946
NM_002364	Melanoma antigen, family B, 2	12.281	<0.001	4.005

Array data were filtered according to Z-score (>3.5 and <−3.5) and p-value (<0.01).

### Molecular differences in cell cycle-related proteins

**Figure 1:**
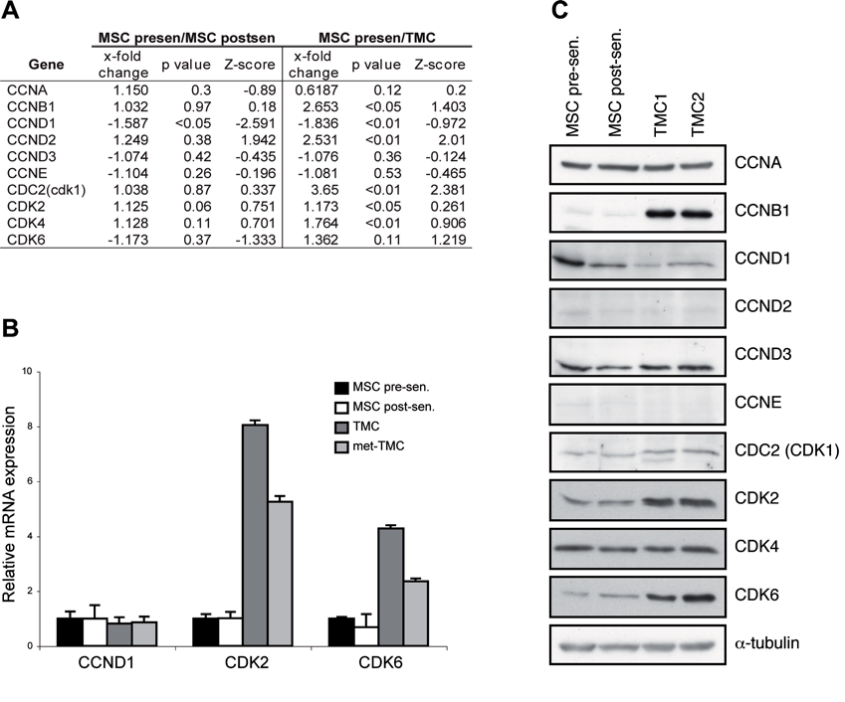
Cell cycle regulation. (A) *x-fold* change, p-value and Z-score of cell cycle regulators expression measured by microarray analysis between pre- and post-senescence MSC and post-senescence MSC and TMC. (B) Relative mRNA expression of Cyclin D1 (CCND1), and cyclin-dependent kinases 2 (CDK2) and 6 (CDK6) in pre- and post-senescence MSC, TMC and met-TMC analyzed by qRT-PCR. (C) Western blot analysis of cell cycle regulator protein expression in pre- and post-senescence MSC and two TMC samples. α-tubulin was used as loading control.

We had reported differences in cell cycle progression in pre- and post-senescence MSC and TMC, including more rapid cell division in TMC [6]. Here we used microarray techniques to explore the cell cycle-related molecular differences in post-senescence MSC and TMC compared to pre-senescence MSC. Compared to pre-senescence MSC, post-senescence MSC showed few differences in cell cycle-related proteins. In contrast, TMC samples showed significant mRNA modulations; Cdk1 and Cdk4 as well as cyclins B1 and D2 were upregulated, whereas cyclin D1 was downregulated (Figure 1A).

We evaluated microarray experiments analyzed by qRT-PCR the expression of some of these genes. No expression difference was found between pre- and post-senescent MSC, while TMC overexpressed Cdk2 and Cdk6 (Figure 1B). In qRT-PCR experiments we also studied met-TMC, a cell line derived of lung metastases generated after s.c. inoculation of TMC in immunodeficient mice (Rubio *et al*, unpublished results).

To determine whether the differences in mRNA levels gave rise to altered protein expression, we compared these samples by western blot. In TMC, cyclin B1, Cdk2 and Cdk6 were upregulated, whereas Cdk1 and Cdk4 remained constant. Cyclin D1 was downregulated from pre-senescence MSC to TMC (Figure 1C).

### Upregulation of DNA repair pathways in transformed MSC

As DNA repair mechanisms are responsible for the bypass of senescence and crisis, as well as for tumor maintenance and progression [14], we analyzed the major proteins linked to these processes. We tested proteins involved in pathways including non-homologous end joining (NHEJ), base excision repair (BER), nucleotide excision repair (NER), mismatch repair (MMR), and homologous recombination (HR).

**Figure 2:**
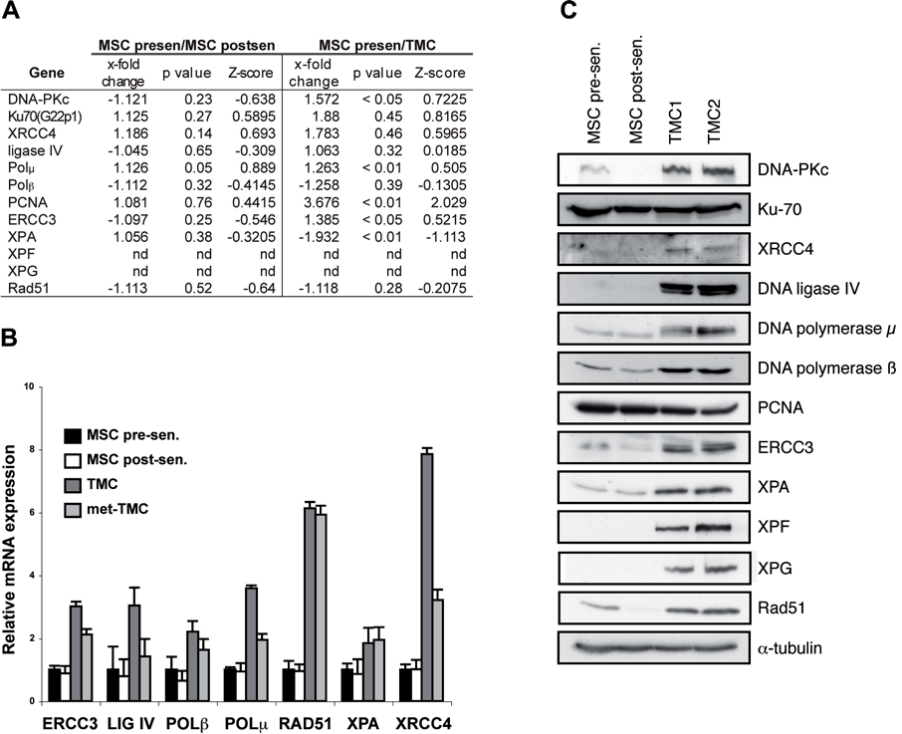
DNA repair regulation. (A) *x-fold* change, p-value and Z-score of DNA repair-related gene expression measured by microarray analysis between pre- and post-senescence MSC and post-senescence MSC and TMC. (B) Relative mRNA expression of ERCC3, DNA ligase IV (LIG IV), DNA polymerase β (POLβ) and μ (POLμ), RAD51, XPA and XRCC4 in pre- and post-senescence MSC, TMC and met-TMC analyzed by qRT-PCR. (C) Western blot analysis of DNA repair-related protein expression in pre- and post-senescence MSC and two TMC samples. α-tubulin was used as loading control.

Microarray analysis showed no mRNA significant differences between pre- and post-senescence MSC (Figure 2A). However protein analysis showed that DNA-PKcs was repressed in post-senescence MSC compared to pre-senescence MSC and a downregulation of Rad51 after senescence bypass, although expression was restored and upregulated after crisis bypass in TMC (Figure 2C).

In contrast, mRNA levels were modulated differentially between TMC and pre-senescence MSC for several DNA repair-associated proteins. Specifically, DNA-PKcs and PCNA were upregulated in TMC, and XPA was downregulated compared to pre-senescence MSC. Although DNA polymerase μ and ERCC3 upregulation were statistically significant, differences in mRNA levels appeared to be minor, based on *x*-fold change and Z-score values (Figure 2A).

We performed qRT-PCR experiments to validate microarray results. No modulation of expression of DNA repair-related genes was detected between pre- and post-senescence MSC. In contrast, several of these genes were overexpressed in TMC, such as DNA-PKcs, DNA polymerase μ, RAD51 or ERCC4 (Figure 2B).

We evaluated the correlation between RNA and protein levels by western blot analysis. Proteins were elevated in nearly all DNA repair pathways in TMC compared to pre-senescence MSC. These effects were protein- rather than pathway-specific. MMR was the only pathway that showed no differences between MSC and TMC, although we analyzed only PCNA in this pathway (Figure 2C).

### Changes in tumor suppressors and oncogenes expression related with MSC transformation

**Figure 3:**
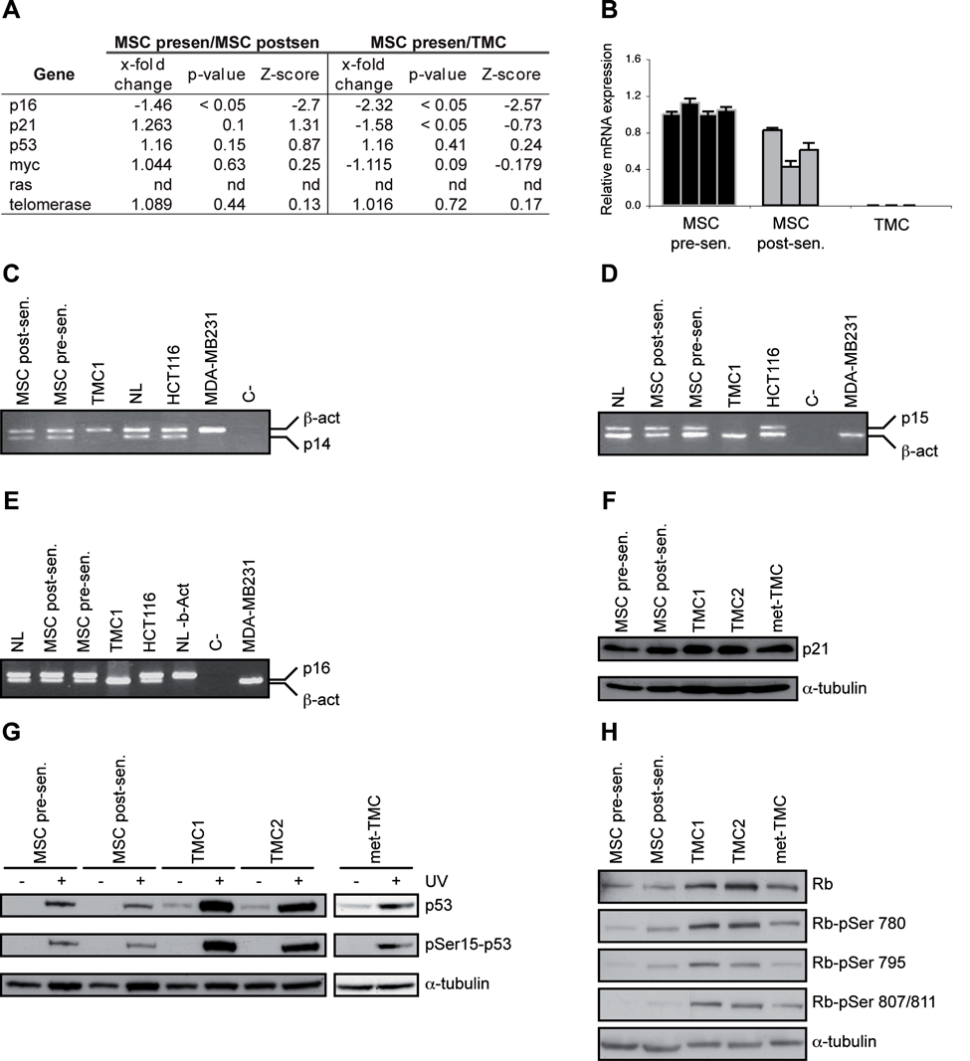
Regulation of oncogenes and tumor suppressor genes. (A) *x-fold* change, p-value and Z-score of oncogenes and tumor suppressor genes measured by microarray analysis between pre- and post-senescence MSC and post-senescence MSC and TMC. (B) Relative mRNA expression of p16 analyzed by qRT-PCR in different samples of pre-senescence MSC (n = 4), post-senescence MSC (n = 3) and TMC (n = 3). (C) Homozygous deletion analysis of p14, p15 (D) and p16 (E) genes. β-actin was used as internal PCR control. Control cell lines were normal lymphocytes (NL), HCT116 and MDA-MB231. (F) Western blot analysis of p21 expression in pre- and post-senescence MSC, TMC and met-TMC. α-tubulin was used as loading control. (G) Analysis of p53 activation following UV irradiation of cells. p53 levels and phosphorylation were tested in pre- and post-senescence MSC, in two TMC samples and a sample of met-TMC. α-tubulin was used as loading control. (H) Rb protein levels and phosphorylation tested in pre- and post-senescence MSC, two samples of TMC and a met-TMC sample. α-tubulin was used as loading control.

To explore the role of tumor suppressor gene inactivation in MSC senescence and crisis bypass, we analyzed p16, p21 and p53. In a microarray screening assay, we found no changes in p21 or p53 levels, whereas MSC expressed less p16 mRNA after senescence bypass (Figure 3A), concurring with reports of a role for p16 in senescence induction [15]. p16 downregulation was more marked when we compared TMC and pre-senescence MSC mRNA levels, and we found slight but significant p21 downregulation. We detected no differences in p53 mRNA (Figure 3A).

To validate the microarray results, we used qRT-PCR to compare mRNA levels among the three cell types. p16 mRNA levels were downregulated in post-senescence MSC, to approximately 60% of the pre-senescence level. We observed no p16 mRNA amplification in TMC samples (Figure 3B). p16 protein is downregulated in post-senescence MSC, and absent in TMC [6], concurring with these results. We analyzed whether complete p16 repression in TMC was caused by promoter methylation, as reported for this tumor suppressor [16], [17] studying the methylation status. Methylation analysis of the Ink4a/Arf promoter showed no methylation (not shown). We used specific DNA-PCR to detect homozygous deletion of p14, p15 and p16, and found no amplification (Figure 3C–E), indicating that the Ink4A/Arf locus is deleted specifically in TMC.

Microarray analysis suggested slight p21 downregulation in TMC compared to pre-senescence MSC, with no difference between pre- and post-senescence MSC. We found no changes in p21 levels in western blot analysis of these populations (Figure 3F). Although p53 pathway defects have been associated with the generation of many tumor types [18], this pathway appeared to be functional in our stem cell transformation model, as p53 was upregulated after UV irradiation and was phosphorylated in all samples tested (Figure 3G). Basal p53 protein levels were also higher in TMC than in pre- or post-senescence MSC.

Finally, we assayed Rb levels and phosphorylation in pre- and post-senescent MSC, two TMC samples and a met-TMC sample. Rb protein levels were upregulated in TMC and met-TMC compared to pre- and post-senescence MSC. Rb phosphorylation increased progressively through the sequence of steps in TMC generation, and was slightly downregulated in met-TMC compared to TMC (Figure 3H).

We compared mRNA regulation differences in the oncogenes c-myc and telomerase in post-senescence MSC and TMC with pre-senescence MSC. We found no differences in oncogene mRNA transcript levels in these populations (Figure 3A). Nonetheless, in a previous publication we have shown that c-myc protein overexpression is linked to senescence bypass and is maintained in TMC; moreover, TMC also have telomerase activity which is not found in pre- or post-senescence MSC [6].

## Discussion

Mesenchymal stem cells can be easily isolated and expanded in culture to generate large numbers of cells for cellular therapies. Human MSC in early passage are safe although stressful conditions (as they are cultured for a long time) can turn them in immortal and occasionally they became tumorogenic [6]. Further research is necessary to understand this process in order to develop better protocols for culture adult stem cells, as it has been demanded recently [19]. Here, we describe several molecular alterations in our spontaneous human MSC transformation model that affect cell cycle regulation, oncogene expression, mitochondrial metabolism, DNA repair mechanisms and inactivation of tumor suppressor genes.

TMC versus pre-senescence MSC array analysis showed that functions with a higher significance are related, as expected, with transformation, genetic disorders and cell death (Table 1). We described previously that post-senescence MSC are non-tumorigenic and their cellular behaviour in culture was very similar to pre-senescence MSC [6]. Interestingly, post-senescence MSC versus pre-senescence MSC array analysis also showed the same functions altered than TMC, although in smaller grade (Table 1), suggesting a pre-tumoral state of pre-senescence MSC.

We observed expression of many untranslated RNAs in MSC concurring with reports which show a large and “silent” mRNA pool in stem cells [20], this could be the reason why, following MSC transformation, we identified more downregulated than upregulated genes in arrays experiments (Figure S1). Comparison of mRNA and protein expression in pre- and post-senescence MSC and in TMC showed variation in RNA and protein regulation. Cyclin D2, Cdk1 and PCNA mRNA were upregulated in TMC compared to MSC, although their protein levels did not change; whereas c-Myc, Cdk2, Cdk6, DNA ligase IV and DNA polymerases mRNA levels remained stable but their protein levels were upregulated. Translational control could thus be important for adult stem cells, and retention of large numbers of silenced transcripts might allow rapid stem cell differentiation to other lineages in response to appropriate stimuli. These data also indicates the limitations of results based on RNA-exclusive analysis of MSC.

Telomerase activity has been found in almost all human tumors but not in adjacent normal cells [21] and maintenance of telomere stability is required for the long-term proliferation of tumor cells [22]. The escape from cellular senescence and thus becoming immortal by activating telomerase is required by most tumor cells for their ongoing proliferation [23]. In our model, during TMC generation these cells acquire a detectable telomerase activity [6]. Telomerase promotes MSC immortalization and, in conjunction with additional events, produces cell transformation [12], [13], [24]. These additional events usually implied an oncogene deregulation.

One of the most important oncogenes involved in MSC transformation is c-myc. In our spontaneous model, senescent and post-senescence MSC, as well as TMC, overexpress c-myc [6]. Consistent with our previous results, data from other groups have shown that c-myc seems to be essential to spontaneously transform MSC [7], [9], [10]. In this regard, Funes et al. used retroviral vectors to introduce human telomerase (TERT), HVP-16 E6 and E7, H-Ras and SV40 small T antigen (ST), individually or in combination, in human MSC. The combination of TERT, E6, E7 and H-Ras did not induce MSC transformation. Only MSC transduced with ST becomes transformed cells [10]. ST inactivates phosphatase 2A, resulting in c-myc stabilization [25], suggesting that c-myc might be necessary to transform MSC.

We explored DNA repair mechanisms to elucidate their role in MSC transformation. Post-senescent MSC showed downregulation of DNA-PKcs, ERCC3 and Rad51 proteins, each of which is associated to a distinct DNA repair pathway. Extremely restricted clonal selection takes place during cell crisis, and only cells with functional DNA repair mechanisms would continue to grow. TMC have a higher metabolic rate and divide more rapidly than pre- or post-senescence MSC, with a consequent increase in DNA damage. Proteins that participate in DNA repair are upregulated in TMC compared to MSC; this, together with telomere length maintenance, could permit cell survival, despite oxidative damage to DNA and be responsible for TMC karyotype stabilization. Recently it has been published the dependency on oxidative phosphorylation during MSC transformation [11]. We have not detected statistically significant changes of these genes in our microarray experiments (Table S1), although potential pathways leading to changes in post-senescence MSC and TMC revealed change in stress, toxic events and mitochondrial metabolism pathways (Table 2). The definitive role of mitochondrial respiration on spontaneous MSC transformation remains to be investigated.

A chromosome 5 alteration and a (3;11) translocation are recurrent, stable features of *in vitro* cultured TMC [6]. The telomerase gene map to human chromosome 5, suggests that it is activated by internal amplification of this chromosome in TMC. Chromosome 11 alterations are recurrent in tumors [26]. Although we did not detect a target gene in the 3;11 translocation in our model, genes involved in cell transformation are likely to be located in this region [27]–[29].

As tumor suppressor genes are major targets in neoplastic transformation, we analyzed their expression in these cells. The tumor suppressor Rb is implicated in several cancer types [18]. In our model of MSC transformation, Rb protein levels are upregulated progressively, and Rb is inactivated by a phosphorylation mechanism in TMC, as described [30]. In addition of Rb, loss of p53 function is common in many tumor types [18], but this pathway appeared to be functional in our model, as p53 was upregulated and phosphorylated in UV-irradiated cells. We observed higher basal p53 levels in TMC than in MSC, even when they had not been exposed to UV irradiation. In TMC, p16 mRNA and protein were entirely absent, and the Ink4a/Arf locus had been deleted. The increase in basal p53 may thus be due to stabilization by the ubiquitin protein ligase MDM2, due to the lack of p16 [31]. Identical results, p16 locus deletion and normal p53 activity, was detected in telomerase-immortalized human MSC [32]. The results suggest that p16 inhibition is essential for TMC generation, as is the case for human malignancies including glioblastoma, melanoma, pancreatic adenocarcinoma, non-small cell lung cancer, bladder carcinoma and oropharyngeal cancer, where this tumor suppressor is frequently lost [33].

**Figure 4:**
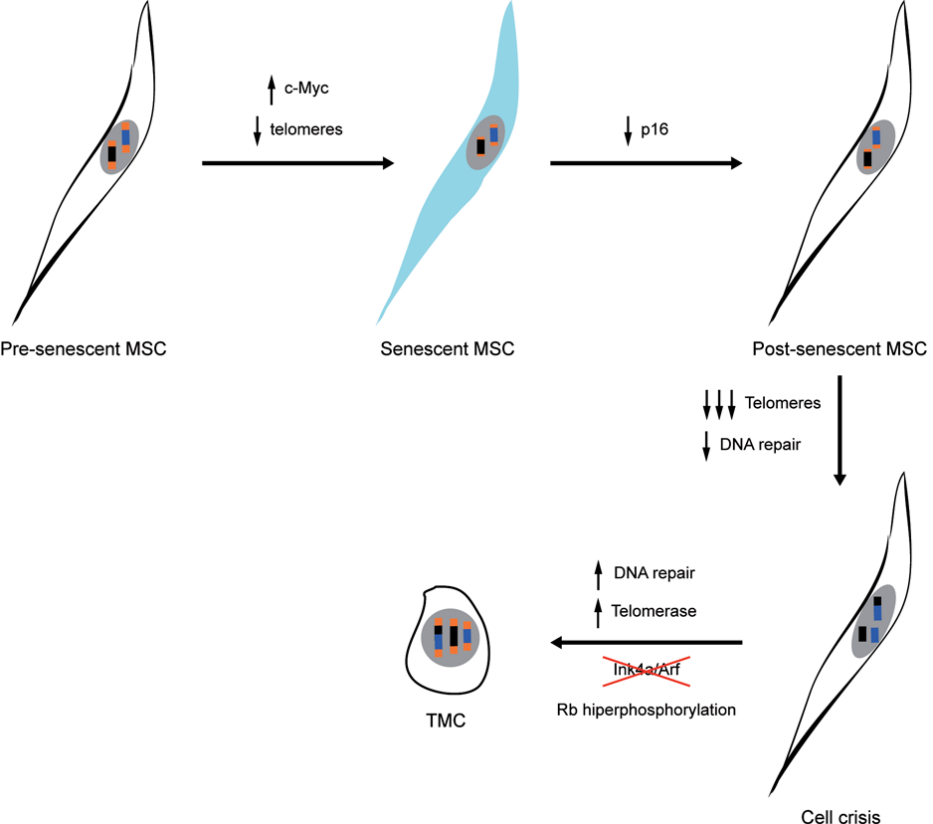
Model of spontaneous human MSC transformation. Sequence of steps: morphologic, karyotypic, and molecular alterations during TMC generation. Black and blue boxes represent chromosomes, orange boxes represent telomeres.

Finally, we propose a two-stage model in which a mesenchymal stem cell becomes a tumor cell (Figure 4). The first step, the senescence bypass or M1 phase, is associated with c-myc overexpression and p16 repression; many DNA repair proteins are subsequently downregulated. Telomere shortening provokes the cell crisis phase, or M2, in which cells undergo stringent selection. TMC then upregulate many DNA repair proteins, which may be necessary for crisis bypass. Finally, escape from crisis is associated with telomere stabilization, Rb hyperphosphorylation and p16 deletion that seems to be essential to promote transformation [33], [34]. TMC also upregulate many DNA repair proteins, which may be necessary for crisis bypass. These levels are maintained in TMC and could permit cell survival, despite oxidative damage to DNA.

The essential steps in TMC generation described here are basically in agreement with results of other authors working in MSC transformation [7]–[11], [32] and these alterations are very similar to molecular changes associated with transformation of other cell types. In epithelial cells, spontaneous immortalization of human keratinocytes exhibited a small number of chromosomal aberrations, reduced p^16INK4a ^mRNA, elevated telomerase activity and functionally normal p53 [35]. Immortalization of human prostate cells by c-myc stabilizes telomere length through up-regulation of TERT expression and lack Rb/p16^INK4a^ checkpoint, being easily transformed [36]. In mesodermic cells, fibroblast cell lines immortalized either spontaneously or radio-chemically induced maintaining their telomerase activity, displayed loss of expression of p16^INK4a^ and hyperphosphorylation of Rb [37]. Telomerase-immortalized human fibroblast revealed overexpression of the c-myc and Bmi-1 oncogenes, as well as loss of p14^ARF^ expression [38], [39], while overexpression of c-myc immortalizes freshly isolated human foreskin fibroblasts displayed a marked decrease in expression of p14^ARF^ [40].

In sum, all these evidences strongly suggest that cells with a mesodermal origin could require a common sequence of oncogenic events to become a tumor cells. How these processes are coordinated or associated with the critical cell evolution/selection revealed in the culture [6] remains to be studied in deep. In addition, the cause/consequence relationship of this molecular signature with the recently characterized mesenchymal to epithelial transition (Rubio, D. et al, in press) or other potentially involved mechanisms remains also to be determined.

## Materials and Methods

### Isolation of MSC and cell culture

MSC were isolated as described [6]. Briefly, adipose tissue from non-oncogenic patients were digested with collagenase P and cultured (37°C, 5% CO_2_) in MSC medium (DMEM plus 10% FCS, 2 mM glutamine, 50 µg/ml gentamycin) and passaged when they reached 85% confluence. TMC and met-TMC were cultured under the same conditions.

### Microarray labeling

Total RNA was isolated from four biological replicates of pre- and post-senescence MSC and from TMC using TriReagent Solution (Sigma) following manufacturer's instructions. RNAs were purified with MegaClear (Ambion) and integrity confirmed using the Agilent 2100 Bioanalyzer (Agilent Technologies). Total RNA (1.5 µg each) was amplified using the Amino Allyl MessageAmp aRNA kit (Ambion); we obtained 15–60 µg of amino-allyl amplified RNA (aRNA). Mean aRNA size was 1500 nucleotides, as measured using the Agilent 2100 Bioanalyzer. For each sample, 2.5 µg of aRNA was labeled with one aliquot of Cy3 or Cy5 Mono NHS Ester (CyDye Post-labeling Reactive Dye, Amersham) and purified with the Amino Allyl MessageAmp aRNA kit. Cy3 and Cy5 incorporation were measured using 1 µl probe in a Nanodrop spectrophotometer (Nanodrop Technologies). For each hybridizations, 80–100 pmol of Cy3 and Cy5 probes were mixed, dried by speed-vacuum, and resuspended in 9 µl RNase-free water. Labeled aRNA was fragmented by adding 1 µl of 10× fragmentation buffer (Ambion) and incubating (70°C, 15 min). The reaction was terminated with 1 µl stop solution (Ambion).

### Slide treatment and hybridization

Slides containing 22,102 annotated genes corresponding to the Human 70-mer oligo library (V2.2) (Qiagen-Operon) were obtained from the Genomics and Microarray Laboratory (Cincinnati University). Information on printing and the oligo set can be found at http://microarray.uc.edu. Slides were prehybridized (42°C, 45–60 min) in 6× SSC, 0.5% SDS and 1% BSA, then rinsed 10 times with distilled water. Fragmented Cy5 and Cy3 aRNA probes were mixed (80–100 pmol of each label) with 10 µg PolyA (Sigma) and 5 µg Human Cot-DNA (Invitrogen) and dried in a speed-vacuum. Each probe mix was resuspended in 60 µl of hybridization buffer (50% formamide, 6× SSC, 0.5% SDS, 5× Denhardt's solution). Probes were denatured (95°C, 5 min) and applied to the slide using a LifterSlip (Erie Scientific). Slides were incubated (48°C, 16 h) in hybridization chambers (Array-It; Telechem International) in a water bath. After incubation, slides were washed twice with 0.5× SSC, 0.1% SDS (5 min each), three times with 0.5× SSC (5 min) and finally in 0.05× SSC (5 min), then dried by centrifugation (563 g, 1 min). Images from Cy3 and Cy5 channels were equilibrated and captured with an Axon 4000B scanner and spots quantified using GenePix 5.1 software.

Four independent biological replicates were “dye swapped” and studied (8 hybridizations). Data were analyzed using Almazen software. Each replicate was lowess-normalized and the log ratios merged with the corresponding standard deviation and z-score. We obtained adjusted p-values using limma by FDR [40]. Differentially expressed genes were selected by filtering signal intensity (>64), z-score (>3.5 or <−3.5) and p-value (<0.01).

### Pathways analysis

By using Ingenuity Pathways Analysis (IPA), potential pathways leading to changes in MSC-postsenescence and TMC were created. This web-delivered application reveals relevant networks by comparing gene expression data with known pathways and interactions between genes (http://www.ingenuity.com). The filtered expression data set for MSC-postsenescence and TMC regulated genes were uploaded as tab-delimited text into IPA for generating biological networks. Each gene identifier was mapped to its corresponding object in the Ingenuity Pathways Knowledge Base. This software assigned a score for all networks that were ranked on the probability that a collection of genes equal to or greater than the number in a network could be achieved by chance alone (a score of 2 represents a 99% confidence level, and 3 a 99.9%). Biological functions are then calculated an assigned to each network

### Quantitative real-time PCR (qRT-PCR)

cDNA was generated from 100 ng of total RNA using the High Capacity cDNA Archive Kit (Applied Biosystems) in a 10 µl final reaction volume. Real-time PCR reactions were performed in triplicate using two dilutions (1/50, 1/500; 3 µl/well) of each cDNA, 1× TaqMan Assay-On-Demand (Hs00233365_m1, Cdkn2a; Hs00195591_m1, snail; Hs00161904_m1, slug) or primers described in Table S2, 1× SYBR Green PCR Master Mix or 1× TaqMan Universal PCR Master Mix (Applied Biosystems) in a volume of 8 µl in 384-well optical plates, or using Universal ProbeLibrary (Roche). PCR reactions were run on an ABI Prism 7900HT (Applied Biosystems) and SDS v2.2 software was used to analyze the results with the Comparative Ct Method (ΔΔCt).

### Western blot

Cell extracts were fractionated in 6%–15% SDS-PAGE, followed by transfer to PVDF membranes. We used antibodies to cyclins A clone E23 (1/200), D1 DCS-11 (1/1000), and D2 DCS-3.1 (1/1000) from Labvision; cyclin B1 sc-595 (1/200), cyclin D3 sc-182 (1/200), cdc2 sc-747 (1/200), cdk2 sc-163 (1/200), cdk4 sc-260 (1/200) and cdk6 sc-177 (1/200) were from Santa Cruz Biotechnology. DNA-PKcs Ab-1 (1/200) was from Calbiochem, and Ku-70 sc-9033 (1/200), XRCC4 sc-8285 (1/200), DNA ligase IV sc-11748 (1/200) were from Santa Cruz Biotechnology. We also used anti-DNA polymerase μ [25], -PCNA Ab-1 (Calbiochem, 1/100), and -DNA polymerase β Ab-1 (1/500), -ERCC1 Ab-2 (1/200), -XPA Ab-1 (1/200), -XPF Ab-1 (1/200), and -XPG Ab-1 (1/200) from Labvision, -Rad-51 (Pharmingen, 1/5000), -p21 sc-397 (Santa Cruz Biotechnology 1/1000), -p53 DO-1 (Merck, 1/1000), and -Rb (1/2000, overnight, 4°C), -phospho Ser 780-Rb (1/1000, overnight, 4°C), -phospho Ser 795-Rb (1/1000, overnight, 4°C), -phospho Ser 807/811-Rb (1/1000, overnight, 4°C) from Cell Signalling. We used anti-tubulin 9026 (Sigma, 1/5000). Incubation was 1 h at room temperature unless otherwise specified, followed by peroxidase-labelled goat anti-mouse, goat anti-rabbit or rabbit anti-goat antibody (Dako, 1/2000, 1 h, RT). Blots were developed using ECL (Amersham).

### p53 activation assay

We induced p53 upregulation and activation in UV-irradiated pre- and post-senescence MSC, TMC, and met-TMC (15 JU/m^2^). Extracts were collected 18 h after irradiation and used in western blot with anti-p53 or -phospho Ser15-p53 antibodies. α-tubulin was used as control.

### Analysis of p16ink4a, p15ink4b and p14ARF CpG island methylation status

We determined DNA methylation patterns in the CpG islands of p16ink4a, p15ink4b and p14ARF tumor suppressor genes by chemical conversion of unmethylated, but not methylated, cytosine to uracil, followed by methyl-specific PCR (MSP) amplification using primers specific for methylated or modified unmethylated DNA [41], [42]. Placental DNA treated *in vitro* with Sss I methyltransferase was used as positive control, and DNA from normal lymphocytes as negative control for methylated alleles. Each PCR sample (12 µl) was separated in non-denaturing 6% polyacrylamide gels, ethidium bromide-stained, and visualized with UV illumination. Promoter methylation status of these genes was verified by bisulfite genomic sequencing of CpG islands. Both strands were sequenced. Primers for bisulfite genomic sequencing and methylation-specific PCR were designed according to genomic sequences around presumed transcription start sites of the genes studied. Primer sequences and PCR conditions for methylation analysis are available on request.

### Bisulfite treatment

Genomic DNA was EcoRI-digested to shear DNA and achieve complete chemical conversion after bisulfite treatment. Sodium bisulfite conversion of genomic DNA (1 µg) was performed as described [41], [42], with modifications. Briefly, NaOH was added to denature DNA (0.3 M final concentration) and incubated (15 min, 37°C). Fresh bisulfite solution (2.5 M sodium metabisulfite and 125 mM hydroquinone, pH 5.0) was added to each sample, and incubation continued (16 h, 50°C, in the dark). Modified DNA was purified using Wizard DNA purification resin (Promega) and eluted in water at 60°C. After desulfonation with NaOH (0.3 M final concentration; 10 min, 37°C), isolation was continued with 0.3 volume of 10.5 M ammonium acetate, followed by incubation (5 min, RT). Modified DNA was precipitated using 2.5 volumes of 100% ethanol and glycogen (5 mg/ml) as a carrier. The pellet was washed with 70% ethanol, dried, and eluted in distilled water.

### Homozygous deletion analysis

We analyzed fragments of the p16INK4a-E1α, E2, p14ARF-E1β, and p15INK4b genes as described [16] to detect homozygous deletion in TMC. Comparative multiplex PCR was performed as described [43] to analyze each gene locus, using the β-actin fragment as internal control. Normal lymphocytes (NL) were used as negative control of tumor suppressor gene methylation, HCT116 (colorectal cancer line) as positive control of Ink4a/Arf locus methylation and MDA-MB231 (mammary adenocarcinoma) as control of Ink4a/Arf locus deletion.

## Supporting Information

Figure S1 Comparison of mRNA differences between pre- and post-senescence MSC and in TMC. Microarray analysis pattern of overall mRNA differences between pre- and post-senescence MSC (A), and pre-senescence MSC and TMC (B). MA plots are shown, being A: log-ratio of two expression intensities vs. M: the mean log-expression of the two.
(4.09 MB TIF)

Table S1 Main mRNA differences between pre-senescence MSC and TMC focused in genes implicated in bioenergetic pathways.
(0.11 MB DOC)

Table S2 Primers used for q-RT-PCR analysis with Universal ProbeLibrary protocol.
(0.04 MB DOC)
