# Supplementary figures and images for: Molecular Characterization of Spontaneous Mesenchymal Stem Cell Transformation
Source: PLoS One. 2008 Jan 2;3(1):e1398. doi: 10.1371/journal.pone.0001398 (PMC2151133; doi:10.1371/journal.pone.0001398)

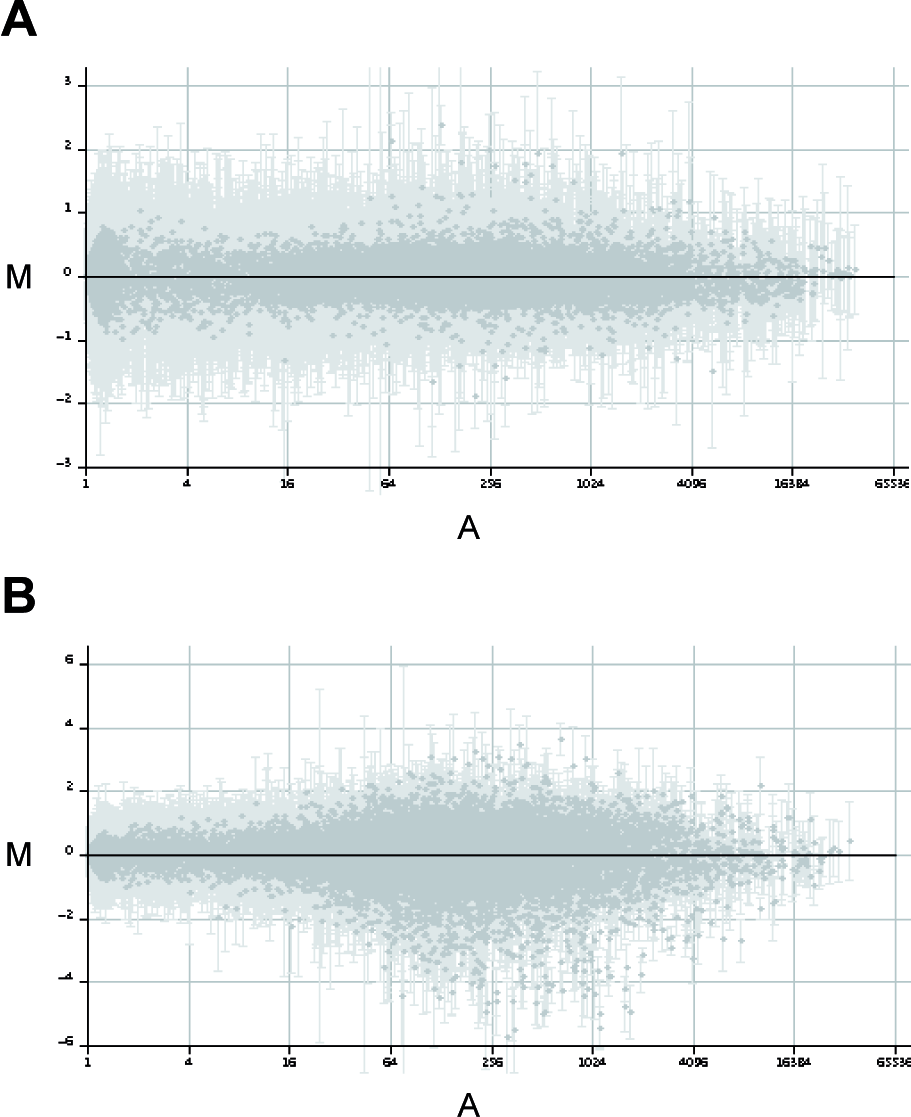

Supplement: Figure S1 — Comparison of mRNA differences between pre- and post-senescence MSC and in TMC. Microarray analysis pattern of overall mRNA differences between pre- and post-senescence MSC (A), and pre-senescence MSC and TMC (B). MA plots are shown, being A: log-ratio of two expression intensities vs. M: the mean log-expression of the two. (4.09 MB TIF) [file pone.0001398.s001.tif]
