# Supplementary material for: Molecular Characterization of Spontaneous Mesenchymal Stem Cell Transformation
Source: PLoS One. 2008 Jan 2;3(1):e1398. doi: 10.1371/journal.pone.0001398 (PMC2151133; doi:10.1371/journal.pone.0001398)
Supplement: Table S1 — Main mRNA differences between pre-senescence MSC and TMC focused in genes implicated in bioenergetic pathways. (0.11 MB DOC) [file pone.0001398.s002.doc]

| **Genbank Acc Nº** | **Gene Name** | **x-fold change** | **p-value** | **z-score** |
| --- | --- | --- | --- | --- |
| AB051452 | Aconitase 2 (ACO2) | 1.097 | 0.291 | 0.323 |
| NM_001124 | Adrenomedullin (ADM) | 2.467 | < 0.001 | 1.886 |
| NM_000034 | Aldolase A (ALDOA) | -1.376 | 0.028 | -0.510 |
| NM_000035 | Aldolase B (ALDOB) | -1.204 | 0.106 | -0.676 |
| NM_005165 | Aldolase C (ALDOC) | -1.903 | 0.018 | -1.117 |
| NM_016109 | Angiopoietin-like 4 (ANGPTL4) | -2.460 | 0.003 | -3.468 |
| BC010106 | Citrate synthase (CS) | -1.294 | 0.080 | -0.413 |
| NM_003467 | Chemokine receptor 4 (CXCR4) | 1.411 | 0.025 | 0.947 |
| AK057299 | Dihydrolipoamide S-acetyltransferase (DLAT) | 1.643 | 0.002 | 0.913 |
| NM_000108 | Dihydrolipoamide dehydrogenase (DLD) | 1.292 | 0.007 | 0.420 |
| NM_001428 | Enolase 1 (ENO1) | 1.076 | 0.683 | 0.117 |
| NM_014355 | Enolase alpha (ENO1B) | 1.016 | 0.869 | 0.038 |
| NM_001975 | Enolase 2 (ENO2) | 1.090 | 0.515 | 0.267 |
| NM_001976 | Enolase 3 (ENO3) | -1.075 | 0.380 | -0.136 |
| NM_000507 | Fructose-1.6-bisphosphatase 1 (FBP1) | 1.041 | 0.479 | 0.117 |
| NM_003837 | Fructose-1.6-bisphosphatase 2 (FBP2) | 1.163 | 0.198 | 0.528 |
| NM_000143 | Fumarate hydratase (FH) | 1.559 | 0.017 | 0.711 |
| NM_000151 | Glucose-6-phosphatase (G6PC) | 1.218 | 0.363 | 0.788 |
| BC006342 | Glucose phosphate isomerase (GPI) | 1.379 | 0.001 | 0.771 |
| NM_013332 | Hypoxia-inducible protein 2 (HIG2) | 2.263 | < 0.001 | 2.081 |
| NM_033500 | Hexokinase 1 (HK1) | 1.019 | 0.849 | 0.030 |
| Z46376 | Hexokinase 2 (HK2) | 1.070 | 0.680 | 0.264 |
| NM_002115 | Hexokinase 3 (HK3) | 1.089 | 0.337 | 0.320 |
| NM_005896 | Isocitrate dehydrogenase 1 (IDH1) | 1.092 | 0.340 | 0.143 |
| NM_002168 | Isocitrate dehydrogenase 2 (IDH2) | 1.247 | 0.339 | 0.461 |
| NM_005530 | Isocitrate dehydrogenase 3 (IDH3A) | 1.177 | 0.061 | 0.414 |
| NM_006899 | Isocitrate dehydrogenase 3 (IDH3B) | 1.147 | 0.052 | 0.219 |
| NM_004135 | Isocitrate dehydrogenase 3 (IDH3G) | -1.104 | 0.270 | -0.367 |
| NM_005566 | Lactate dehydrogenase A (LDHA) | -2.035 | 0.035 | -1.152 |
| BC008952 | Lactate dehydrogenase B (LDHB) | 2.351 | 0.002 | 1.368 |
| NM_002301 | Lactate dehydrogenase C (LDHC) | 1.088 | 0.443 | 0.231 |
| NM_033195 | Lactate dehydrogenase A -like (LDHL) | -1.202 | 0.344 | -0.695 |
| NM_005917 | Malate dehydrogenase 1(MDH1) | 1.325 | 0.078 | 0.450 |
| NM_005918 | Malate dehydrogenase 2 (MDH2) | 1.164 | 0.355 | 0.329 |
| AF334710 | pyruvate dehydrogenase kinase 4 (PDK4) | -8.534 | < 0.001 | -4.138 |
| NM_002541 | Oxoglutarate dehydrogenase (OGDH) | -1.343 | 0.008 | -0.502 |
| NM_000920 | Pyruvate carboxylase (PC) | -1.586 | 0.030 | -0.932 |
| NM_000284 | Pyruvate dehydrogenase (PDHA1) | 1.228 | 0.438 | 0.950 |
| NM_005390 | Pyruvate dehydrogenase (PDHA2) | -1.157 | 0.180 | -0.543 |
| NM_000925 | Pyruvate dehydrogenase (PDHB) | 1.109 | 0.440 | 0.404 |
| NM_002610 | Pyruvate dehydrogenase kinase (PDK1) | 1.066 | 0.734 | 0.224 |
| NM_002611 | Pyruvate dehydrogenase kinase (PDK2) | 1.210 | 0.224 | 0.559 |
| NM_005391 | Pyruvate dehydrogenase kinase (PDK3) | 1.509 | 0.014 | 0.891 |
| NM_002612 | Pyruvate dehydrogenase kinase (PDK4) | -1.188 | 0.390 | -0.569 |
| NM_018444 | Pyruvate dehydrogenase phosphatase (PDP) | 1.264 | 0.101 | 0.950 |
| NM_003477 | Pyruvate dehydrogenase complex (PDX1) | 1.579 | 0.033 | 1.512 |
| NM_002626 | Phosphofructokinase. liver (PFKL) | -1.899 | 0.008 | -1.027 |
| NM_000289 | Phosphofructokinase. muscle (PFLM) | 2.015 | < 0.001 | 1.320 |
| NM_002627 | Phosphofructokinase. platelet (PFKP) | -1.232 | 0.075 | -0.335 |
| NM_002629 | Phosphoglycerate mutase 1 (PGAM1) | -1.151 | 0.367 | -0.225 |
| NM_000290 | Phosphoglycerate mutase 2 (PGAM2) | 1.049 | 0.643 | 0.159 |
| NM_000291 | Phosphoglycerate kinase 1 (PGK1) | 1.806 | 0.008 | 0.946 |
| NM_004168 | Succinate dehydrogenase complex. subunit A (SDHA) | 1.150 | 0.507 | 0.566 |
| NM_003000 | Succinate dehydrogenase complex. subunit B (SDHB) | 2.599 | 0.002 | 1.756 |
| NM_003001 | Succinate dehydrogenase complex. subunit C (SDHC) | 1.594 | 0.054 | 1.541 |
| NM_003002 | Succinate dehydrogenase complex. subunit D (SDHD) | 1.742 | 0.039 | 1.937 |
| NM_006516 | Solute carrier family 2. member 1 (SLC2A1) | -1.402 | 0.002 | -0.541 |
| NM_000340 | Solute carrier family 2. member 2 (SLC2A2) | -1.023 | 0.936 | -0.098 |
| NM_006931 | Solute carrier family 2. member 3 (SLC2A3) | -1.071 | 0.480 | -0.175 |
| NM_003850 | Succinate-CoA ligase. ADP-forming (SUCLA2) | 1.485 | 0.003 | 0.653 |
| NM_003849 | Succinate-CoA ligase. GDP-forming (SUCLG1) | 1.051 | 0.841 | 0.259 |
| BC007716 | Succinate-CoA ligase. GDP-forming (SUCLG2) | 1.776 | 0.005 | 1.380 |
| NM_006755 | Transaldolase 1 (TALDO1) | -1.987 | < 0.001 | -1.099 |
| NM_000365 | Triosephosphate isomerase 1 (TPI1) | 2.070 | 0.002 | 1.162 |
| NM_005429 | Vascular endothelial growth factor (VEGF) | 1.490 | < 0.001 | 0.639 |
