# Supplementary material for: Molecular Characterization of Spontaneous Mesenchymal Stem Cell Transformation
Source: PLoS One. 2008 Jan 2;3(1):e1398. doi: 10.1371/journal.pone.0001398 (PMC2151133; doi:10.1371/journal.pone.0001398)
Supplement: Table S2 — Primers used for q-RT-PCR analysis with Universal ProbeLibrary protocol. (0.04 MB DOC) [file pone.0001398.s003.doc]

| Gene | Primer 5’ | Primer 3’ |
| --- | --- | --- |
| 28S | tgccatggtaatcctgctca | cctcagccaagcacatacacc |
| CCND1 | gaagatcgtcgccacctg | gacctcctcctcgcacttct |
| CDK2 | cctcctgggctgcaaata | cagaatctccagggaataggg |
| CDK6 | tgatcaactaggaaaaatcttggac | ggcaacatctctaggccagt |
| ERCC3 | gtggtggctggggaattt | agaactcatggtgccaaagc |
| DNA ligase IV | acagaggtaacggagcttgc | gatgcaacagtttgtgaagtttg |
| DNA polymerase β | ctcgagttagtggcattggtc | ttttaattccttcatctacaaacttcc |
| DNA polymerase μ | ccccagaaactaacccaaca | gactggggtgctcaggtc |
| RAD51 | tgagggtacctttaggccaga | cactgccagagagaccatacc |
| XPA | cgagtatcgagcggaagc | tttggggctgcttttacatt |
| XRCC4 | ggagaggtaggatccggaag | tggattctgcttatttttctctcc |
